# Phosphatidylserine-binding receptor, CD300f, on macrophages mediates host invasion of pathogenic and non-pathogenic rickettsiae

**DOI:** 10.1128/iai.00059-25

**Published:** 2025-05-01

**Authors:** Oliver H. Voss, Imran Moin, Hodalis Gaytan, Mohammad Sadik, Saif Ullah, M. Sayeedur Rahman

**Affiliations:** 1 Department of Microbiology and Immunology, University of Maryland School of Medicine 200790, Baltimore, Maryland, USA; Washington State University, Pullman, Washington, USA

**Keywords:** *R. typhi*, *R. rickettsii*, *R. montanensis*, CD300f, phosphatidylserine, phagocytosis, macrophages, bacterial host dissemination

## Abstract

Some arthropod-borne obligate intracellular rickettsiae are among the most virulent human pathogens. *Rickettsia* species modulate immune (e.g., macrophages; MΦ) and non-immune cell (e.g., endothelial cells) responses to create a habitable environment for host colonization. MΦ play a crucial role in either terminating an infection at an early stage or succumbing to bacterial replication and colonization. However, our understanding of how *Rickettsia* species invade host cells, including MΦ, remains poorly defined. In this study, we describe a mechanism of host invasion by *Rickettsia* species, involving rickettsial phosphatidylserine (PS), as a ligand, and the CD300f receptor on MΦ. Our data reveal that engulfment of both pathogenic *Rickettsia typhi* (the etiologic agent of murine typhus) and *Rickettsia rickettsii* (the etiologic agent of Rocky Mountain spotted fever) species, as well as the non-pathogenic *Rickettsia montanensis*, is significantly reduced in bone marrow-derived macrophages (BMDMΦ) from CD300f^-/-^ mice, as compared to that of wild-type (WT) animals. Furthermore, our mechanistic analysis suggests bacterial PS as the potential source for the CD300f-mediated rickettsiae engulfment by MΦ. *In vivo* infection studies using WT and CD300f^-/-^ C57BL/6J mice show that CD300f^-/-^ animals are protected against *R. typhi*- or *R. rickettsii*-induced fatal rickettsiosis, which corroborates with the level of the bacterial burden detected in the spleens of the mice. Adoptive transfer studies reveal that CD300f-expressing MΦ are important mediators to control rickettsiosis *in vivo*. Collectively, our findings describe a previously unappreciated role for the efferocytic receptor, CD300f, to facilitate engulfment of rickettsiae within the host.

## INTRODUCTION

Vector-borne diseases represent a serious concern for global public health. In the United States, many vector-borne pathogens, transmitted by hematophagous arthropods, are on the rise, as exemplified by recent outbreaks of *Rickettsia rickettsii*, an etiologic agent of Rocky Mountain Spotted Fever, in Arizona (1) and of *Rickettsia typhi*, an etiologic agent of murine typhus, in California (2) and in Texas (3). Human infection with rickettsiae occurs via infected arthropods (e.g., ticks, mites, and fleas) either through their bite or deposited infected feces onto the host’s dermis and mucosal surfaces. At the inoculation site within the host dermis, rickettsiae encounter tissue-resident immune cells such as macrophages (MΦ), as well as dendritic cells, and then disseminate to endothelial cells (4–6). MΦ play a crucial role in either terminating an infection at an early stage of host invasion, which commonly is the fate of non-pathogenic *Rickettsia* species, or succumbing to bacterial replication and pathogen colonization as well as dissemination to distant organs of host (4–6). However, various key cellular processes of rickettsial obligate lifestyle, which includes (i) internalization by phagocytosis into professional phagocytes (e.g., MΦ) or endothelial cells, (ii) regulation of membrane dynamics and intracellular trafficking, and (iii) evasion of host defenses to establish an intracytosolic replication niche, remain poorly defined and have impaired the development of effective interventions against pathogenic rickettsiae (4, 7). Specifically, our understanding of rickettsiae-induced phagocytosis relies mostly on reports from endothelial cells, leaving the mechanism of rickettsiae engulfment by host defense cells, such as MΦ, mostly unknown. In fact, previous research on endothelial cells has revealed the identity of four host receptors Ku70 (8), α2β1 (9), FGFR1 (10), and Epac1 (11), with limited effects on *Rickettsia* invasion (~40%) when silenced individually, supporting the hypothesis of an alternative receptor (on host)-ligand (on rickettsiae) system for rickettsial engulfment. Yet the precise mechanism(s) remains to be determined.

Phagocytosis of apoptotic cells by MΦ (aka efferocytosis) is critical for maintaining homeostasis by removing apoptotic cells before becoming proinflammatory and immunogenic, causing autoimmune diseases (12, 13). Importantly, the same mechanism can also be hijacked by various pathogens, including viruses and bacteria, to promote host cell invasion (14). In fact, pathogens have been shown to exploit efferocytosis through externalization of “eat-me” signals (e.g., phosphatidylserine [PS]) to gain access to various PS-binding receptors on MΦ, including CD300s (14, 15) to evade host immune surveillance, and to promote their dissemination within the host (14). The CD300 family consists of type I transmembrane cell surface receptors with a single immunoglobulin (Ig)V-like extracellular domain that can transmit either activating or inhibitory signals (13, 16, 17). Specifically, activating receptors have usually short intracellular tails and gain activation potential through binding of adaptor molecules (e.g., DAP12) harboring immunoreceptor tyrosine-based activation motifs. In contrast, inhibitory receptors have immunoreceptor tyrosine-based inhibitory motifs within their intracellular tails. The orthologous mouse family has a variety of names, including CMRF-like molecules (CLM) (17), but for simplicity, herein, we use the human nomenclature for both species. CD300f is predominantly expressed by myeloid cells and is unique as it displays both activating and inhibitory signaling capabilities (13, 17). Moreover, CD300f is a PS-binding receptor, which regulates efferocytosis in professional phagocytes (e.g., MΦ) (13, 17–21). In line with such a unique role, CD300f has also been shown to play an important role in modulating allergic, inflammatory, autoimmune, viral, and bacterial responses. However, the contributing role of PS-binding receptor CD300f during rickettsiae engulfment in MΦ (or other immune defense cells), causing ultimately fatal rickettsiosis, has not been investigated.

Here, we assessed the functional role of CD300f in rickettsial pathogenesis. Accordingly, we determined the phagocytic capability of bone marrow-derived macrophages (BMDMΦ) from wild-type (WT) and CD300f^-/-^ mice infected with *R. typhi*, *R. rickettsii*, or *Rickettsia montanensis*. Our data revealed that engulfment of pathogenic as well as non-pathogenic *Rickettsia* species was reduced in CD300f^-/-^ BMDMΦ as compared to that of WT BMDMΦ. Further mechanistic analysis indicated that the CD300f receptor modulates the phagocytosis of all three *Rickettsia* species, likely via rickettsial PS as ligand. *In vivo* infection studies, using our previously established mouse model of fatal rickettsiosis (22), revealed that CD300f^-/-^ mice (19), but not WT animals, were protected against *R. typhi*- or *R. rickettsii*-induced lethality. Using adoptive transfer studies, we showed that CD300f-expressing MΦ were critical mediators in controlling rickettsial infection *in vivo*. Collectively, our findings describe a previously unappreciated role of the CD300f receptor during rickettsial infection.

## RESULTS

### CD300f modulates engulfment of pathogenic and non-pathogenic rickettsiae

**Fig 1:**
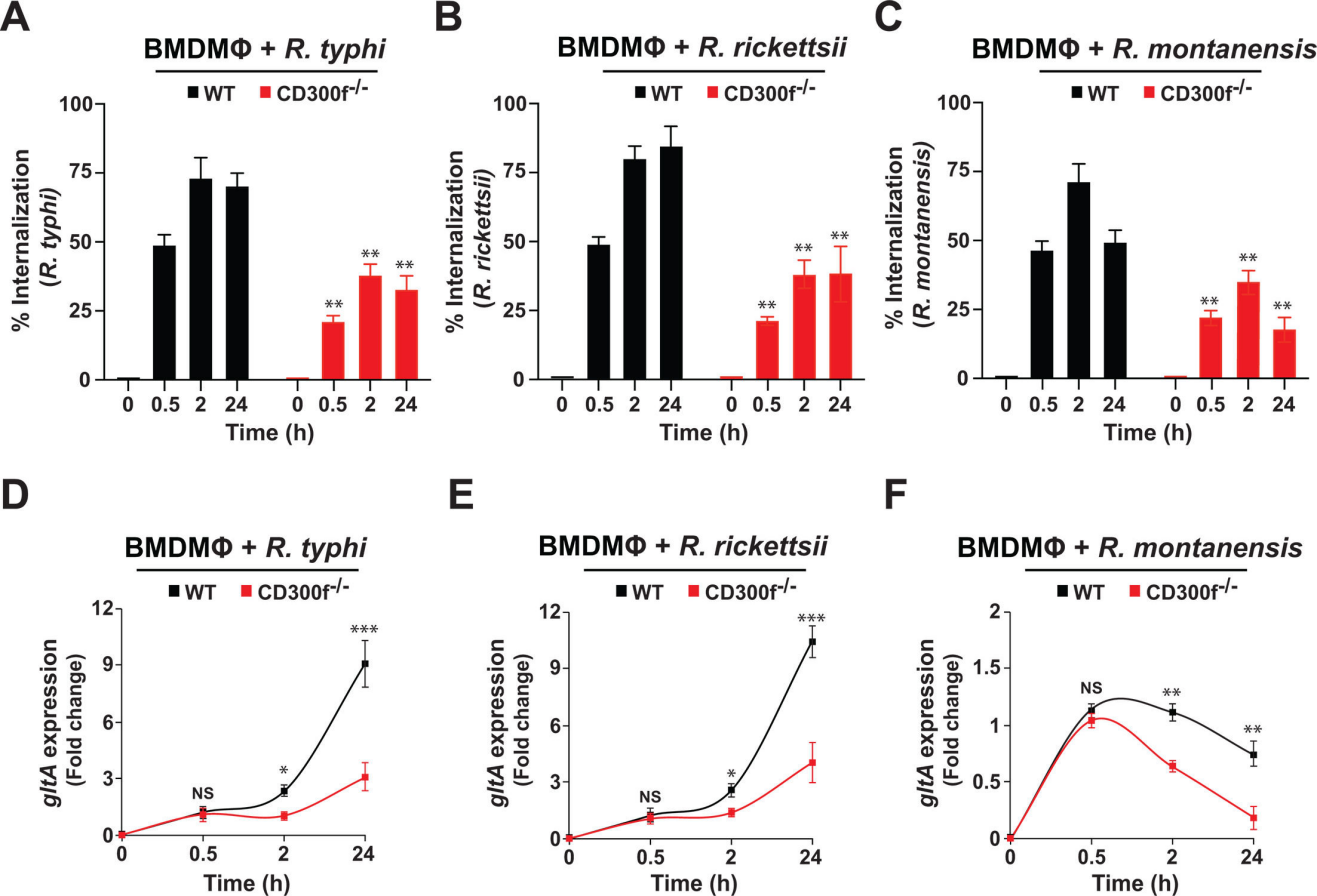
Phosphatidylserine receptor, CD300f, facilitates the engulfment of pathogenic and non-pathogenic rickettsiae. BMDMΦ from WT or CD300f^-/-^ mice were infected with partially purified *R. typhi*, *R. rickettsii*, and *R. montanensis* at an MOI of 20 (0.5 and 2 h) and 5 (24 h). (**A–C**) Rickettsial invasion was monitored by IFA at 0.5, 2, and 24 hpi as described previously (23). Bacterial burdens of WT and CD300f^-/-^ BMDMΦ infected with *R. typhi* (**D**), *R. rickettsii* (**E**), or *R. montanensis* (**F**) were determined at 0.5, 2, and 24 hpi (*n* = 5 per group) by RT-qPCR. RCN of *gltA* expression of rickettsiae was normalized by the expression of the housekeeping host gene, *GAPDH*. Error bars in panels A–F represent the means ± SEM from five independent experiments. NS, nonsignificant; *P ≤ 0.05; **P ≤ 0.01; ***P ≤ 0.005.

Efferocytosis carried out by professional phagocytes, such as MΦ, is critical for maintaining cellular homeostasis by removing large quantities of apoptotic cells before they become proinflammatory and immunogenic, causing the development of autoimmune diseases (12–14). Intriguingly, the same mechanism can also be hijacked by viruses and bacteria to promote their host invasion by utilizing PS externalization to gain access to PS receptors, including Tyro3, Axl, MerTK, TIM1/4, or CD300s (e.g., CD300f) (14, 15). Importantly, MΦ are one of the host defense cell types first encountered by rickettsiae at the site of inoculation. In addition, MΦ are considered to be key mediators either to terminate the infection at the early stage or to allow pathogen colonization and subsequent dissemination throughout the host body (4). However, how rickettsiae gain access into host defense cells, such as MΦ, remains to be investigated. In this study, we test the hypothesis that *Rickettsia* species utilize the PS-binding receptor CD300f to invade and colonize MΦ. In this effort, we determined the invasion of *R. typhi*, *R. rickettsii*, and *R. montanensis* into BMDMΦ, from either WT or CD300f-deficient mice, by immunofluorescent assay (IFA) (23) and observed that CD300f facilitated the internalization of all tested rickettsiae (Fig. 1A through C). To further confirm these data, we evaluated the bacterial burden (measured by *gltA* expression) by employing RT-qPCR. For all three *Rickettsia* species, we found similar results at early time points (Fig. 1D through F); however, *R. montanensis* showed a reduced growth at 2 and 24 h (Fig. 1F), supporting our previous result that *R. montanensis*, but not *R. typhi* and *R. rickettsii*, is cleared by MΦ (22, 24). As the CD300 family is composed of several members (13, 17, 19, 20, 25), we determined the expression of various members of the CD300 family by RT-qPCR in uninfected WT and CD300f-deficient BMDMΦ. The data showed that the lack of CD300f expression did not affect the expression of other CD300 members, including *CD300a*, *CD300b*, *CD300c*, and *CD300d* (Fig. S1A). Of note, the expression level of *CD300c* was significantly lower as compared to all other tested *CD300*s in both WT and CD300f-deficient BMDMΦ (Fig. S1A). Together, our data indicate an important role for CD300f by modulating rickettsiae engulfment in MΦ. To further validate this hypothesis, we evaluated the engulfment of all three *Rickettsia* species using BMDMΦ isolated from WT, CD300f^-/-^, and CD300d^-/-^ (a highly expressed activating receptor in MΦ, Fig. S1A) mice by IFA and RT-qPCR. Our data revealed that, unlike in WT or CD300d^-/-^ BMDMΦ, engulfment and bacterial burden of *R. typhi*, *R. rickettsii*, and *R. montanensis* were significantly reduced in CD300f^-/-^ BMDMΦ (Fig. S1B through G), suggesting that the CD300f receptor plays a key role in the engulfment of pathogenic and non-pathogenic rickettsiae by MΦ.

### Phosphatidylserine is involved in the CD300f-mediated engulfment of rickettsiae

**Fig 2:**
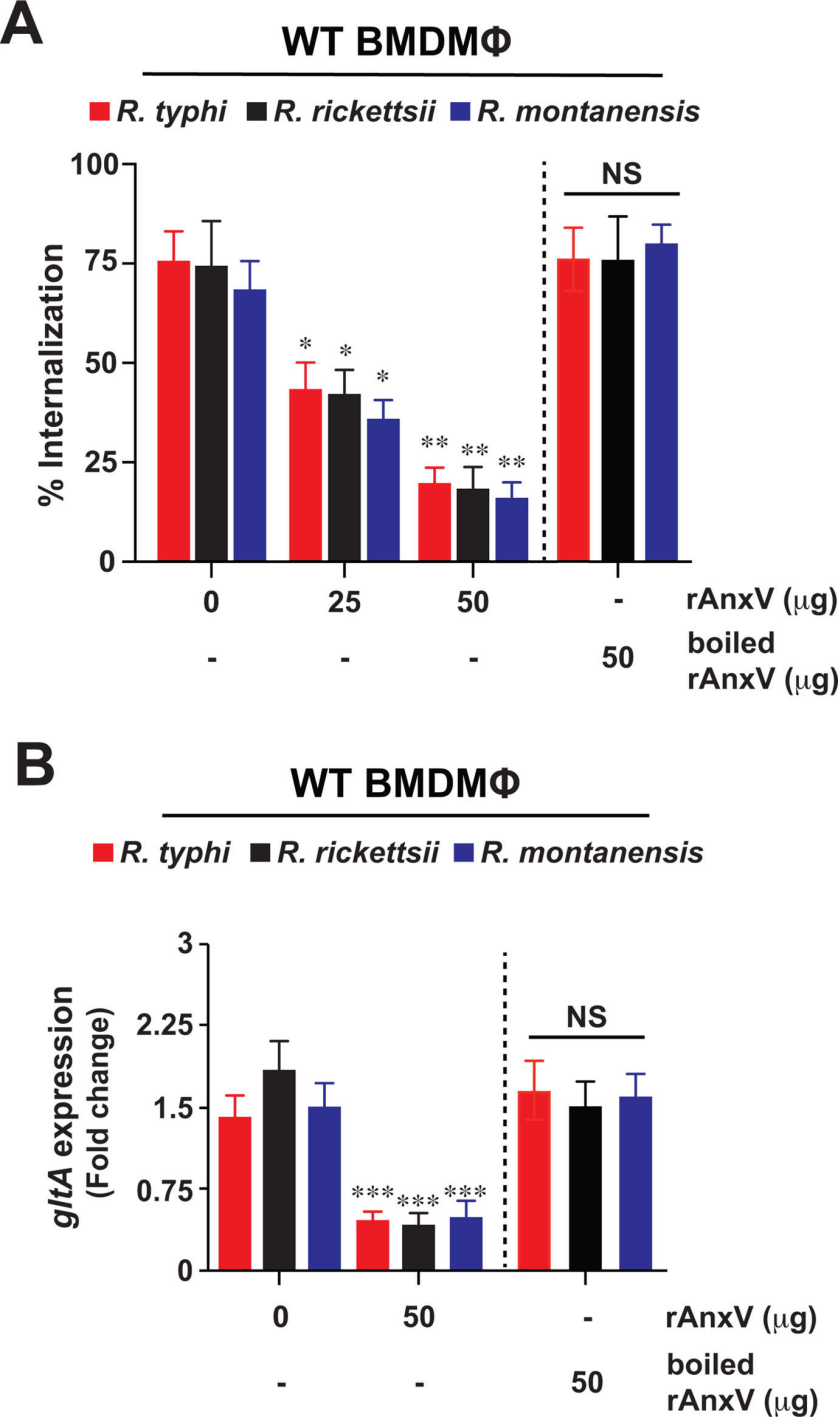
Blocking phosphatidylserine reduces invasion of pathogenic and non-pathogenic rickettsiae in macrophages. Partially purified *R. typhi*, *R. rickettsii*, and *R. montanensis* were pretreated for 0.5 h with various amounts of rAnxV and then utilized to infect BMDMΦ from WT mice. Cells were infected with rickettsiae (untreated or pretreated with rAnxV) for 2 hpi using an MOI of 20. Boiled rAnxV served as a control. (**A**) Rickettsial invasion was monitored by IFA at 2 hpi as described previously (23). (**B**) Bacterial burden of WT BMDMΦ infected with *R. typhi*, *R. rickettsii*, or *R. montanensis* was determined at 2 hpi (*n* = 5 per group) by RT-qPCR. RCN of *gltA* expression of rickettsiae was normalized by the expression of the housekeeping host gene, *GAPDH*. Error bars in panels A and B represent the means ± SEM from five independent experiments. NS, nonsignificant; *P ≤ 0.05; **P ≤ 0.01; ***P ≤ 0.005.

As CD300f primarily functions as a PS-binding receptor to regulate phagocytic events like efferocytosis (13, 18–21, 25), we sought to test the hypothesis that CD300f-mediated engulfment of *Rickettsia* species by MΦ involves rickettsial PS as ligand. In this effort, we incubated partially purified *R. typhi*, *R. rickettsii*, and *R. montanensis* with increasing concentrations of recombinant Annexin V (rAnxV), a molecule known to bind to and inhibit PS-mediated processes like efferocytosis (18, 19, 25). Following incubation, the rAnxV-treated rickettsiae were utilized to infect WT BMDMΦ. Rickettsiae engulfment and burden were evaluated by IFA and RT-qPCR, respectively (22, 23). Our data revealed that pretreatment with rAnxV significantly reduced the engulfment (Fig. 2A) and burden (Fig. 2B) of all three rickettsiae. In addition, experiments using heat-inactivated (HI) rAnxV were employed as specificity control and failed to inhibit the bacterial invasion in MΦ (Fig. 2A and B), indicating that PS is a putative ligand contributing to the rickettsial invasion process.

### Pathogenic and non-pathogenic *Rickettsia* species express phosphatidylserine

**Fig 3:**
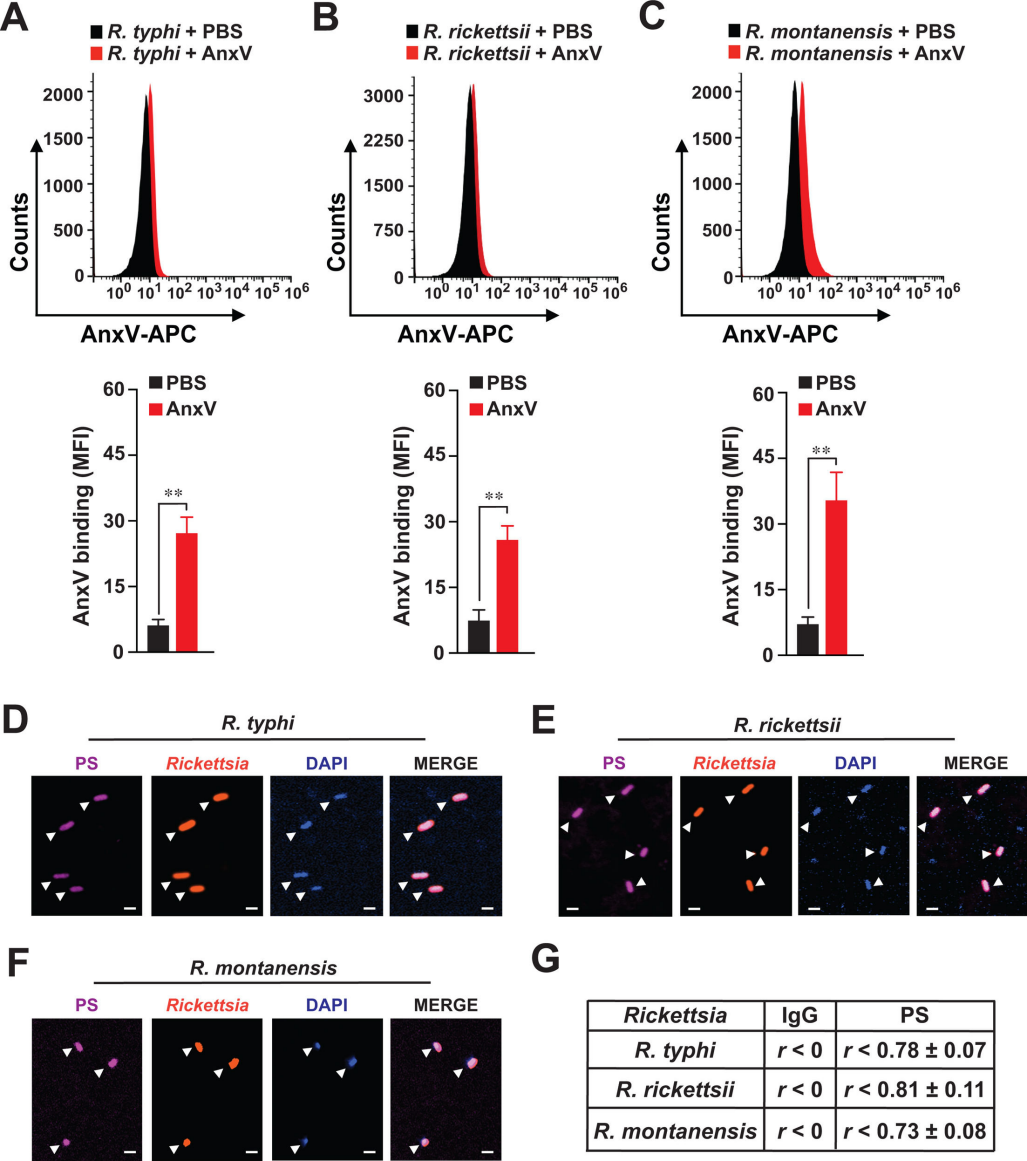
Pathogenic and non-pathogenic rickettsiae express phosphatidylserine. (**A–C**) Partially purified *R. typhi*, *R. rickettsii*, and *R. montanensis* were fixed with 70% ethanol and treated with lysozyme. Bacteria were then incubated with APC-conjugated AnxV for 30 min and acquired using a Becton-Dickinson Accuri C6 machine. Samples were analyzed by the FlowJo software, and fluorescence was expressed as mean fluorescence intensity (MFI). Error bars in panels **A–C** represent the means ± SEM from five independent experiments. NS, nonsignificant; **P ≤ 0.01. (**D–G**) Partially purified *R. typhi*, *R. rickettsii*, and *R. montanensis* were fixed with 4% PFA and analyzed by IFA. Samples were stained with Alexa Fluor-594-conjugated anti-*Rickettsia* (SFG [1:100]; TG [1:500]) Abs as well as Alexa Fluor-647-conjugated anti-PS Ab (1:100). The cell nuclei were stained with DAPI. (**G**) Co-localization between *Rickettsia* and anti-PS or anti-IgG control Ab staining was analyzed using the Coloc 2 plugin Fiji software and was displayed as a Pearson correlation coefficient (*R*) (30); 0 < *r* < 0.39 low correlation; 0.4 < *r* < 0.59 moderate correlation; 0.6 < *r* < 0.79 high correlation; 0.8 < *r* < 1 very high correlation. Bars in panels **D–F**, 1 µm. Approximately 100 bacteria-infected cells were analyzed per condition and time point. Presented images are representative of three independent experiments.

To further elucidate the mechanism of PS-CD300f-mediated engulfment of rickettsiae, we wanted to delineate the source of PS. Detection of PS using fluorescently labeled AnxV or anti-PS antibodies is commonly used to evaluate apoptotic events in eukaryotic cells; however, only a few studies have utilized such approaches to determine PS expression on bacterial cells (26, 27). In fact, leaflets of outer membranes from gram-negative bacteria are highly asymmetric and considered depleted of many phospholipids but enriched in lipopolysaccarides (LPS) suggesting limited accessibility to detect phospholipids on rickettsial membranes (28). To overcome such limitation, we pretreated partially purified rickettsiae with ethanol and lysosome prior to PS staining, as described elsewhere (26). We evaluated the expression of PS of *R. typhi*, *R. rickettsii*, and *R. montanensis* by flow cytometry using allophycocyanin (APC)-conjugated AnxV and showed that all three rickettsiae express PS on the surface (Fig. 3A through C). To address the PS expression within each *Rickettsia* species, we employed IFA as described previously (22, 24) and showed that anti-PS antibody-stained *R. typhi*, *R. rickettsii*, or *R. montanensis* expressed significant levels of PS when compared to IgG-antibody-stained bacteria (Fig. 3D through G; Fig. S2). These data suggest that pathogenic and non-pathogenic *Rickettsia* species express external and internal PS moieties. As our rickettsiae were purified using the glass bead method (29), we were wondering whether the observed PS-mediated phagocytic phenotype would be a technical artifact of the actual purification process. To investigate the extent of potential PS carryover from host cell membrane remnants, we infected WT or CD300f^-/-^ BMDMΦ with purified rickettsiae of different purity levels (partially purified [PP], crudely purified [CP], and sucrose purified [SP]) as described in the Materials and Methods section, and performed phagocytosis studies for various lengths of time. Similar to our data shown in Figure 1, phagocytosis of *R. typhi* (PP), *R. rickettsii* (PP), and *R. montanensis* (PP) was significantly reduced in CD300f^-/-^ BMDMΦ compared to that in WT BMDMΦ (Fig. S3A, D, and G). Intriguingly, phagocytosis studies using either CP (Fig. S3B, E, and H) or SP (Fig. S3C, F, and I) rickettsiae revealed similar internalization kinetics, suggesting that any PS originated from host cell membrane remnants did not alter the observed phagocytic process.

### CD300f exacerbates the pathogenesis of *Rickettsia* infection

**Fig 4:**
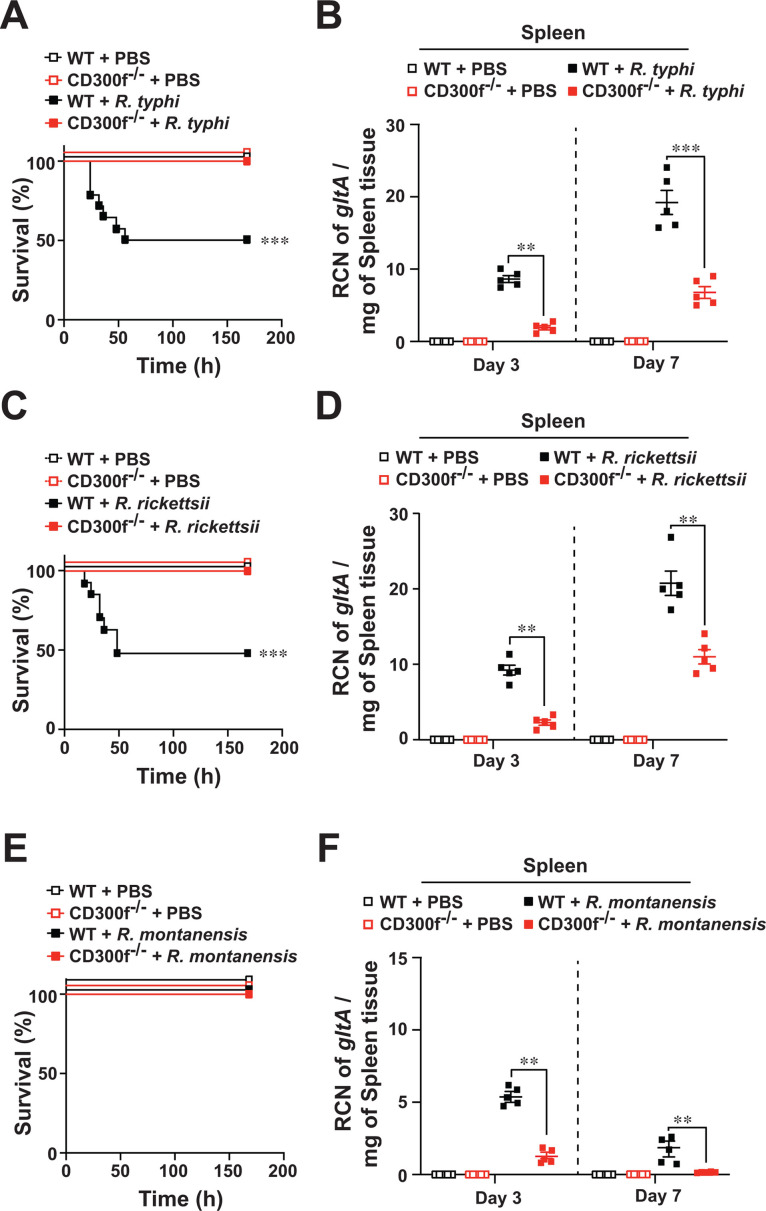
Efferocytic receptor, CD300f, exacerbates the pathogenesis of rickettsiosis. C57BL/6J WT or CD300f^-/-^ mice were injected via tail vein (i*.*v.) with *R. typhi* (**A and B**), *R. rickettsii* (**C and D**), *R. montanensis* (**E and F**), and PBS (10^6^ PFU, *n* = 12 for each treatment). Survival was monitored for 7 days (A, C, and E). Bacterial burden was tested in spleens of *R. typhi*-, *R. rickettsii*-, *R. montanensis*-, or PBS-injected WT and CD300f^-/-^ mice shown in panels B, D, and F by RT-qPCR at days 3 and 7 (*n* = 5 for each treatment). RCN of *gltA* expression of rickettsiae was normalized by the expression of the housekeeping host gene, *GAPDH*. Error bars in panels B, D, and E represent the means ± SEM from five independent experiments. **P ≤ 0.01, ***P ≤  0.005.

Given our findings of PS-CD300f-mediated engulfment of rickettsiae in MΦ, we next tested *in vivo* the role of CD300f in *Rickettsia* colonization by employing our established C57BL/6J WT mouse model of severe (~LD_50_) rickettsiosis (22). In agreement with our published findings, we observed a mortality rate of ~50% in WT mice using *R. typhi* (~68 hpi) and *R. rickettsii* (~50 hpi) (Fig. 4A and C). No signs of lethality in *R. montanensis*-infected mice were observed (Fig. 4E), which is in agreement with our previous report (22). Additionally, *R. typhi*-, *R. rickettsii*-, or *R. montanensis*-infected CD300f^-/-^ mice showed no signs of lethality during the course of infection (Fig. 4A, C, and E). We confirmed the successful infection of WT or CD300f^-/-^ mice with the tested *Rickettsia* species at day 3 postinfection by evaluating the bacterial burdens in splenic tissues (Fig. 4B, D, and F). Of note, bacterial burdens of *R. typhi*-, *R. rickettsii*-, and *R. montanensis*-infected WT mice were significantly higher than that of rickettsiae-infected CD300f^-/-^ mice at days 3 and 7 (Fig. 4B, D, and F). This correlated with the differences observed in the spleen weights of the rickettsiae-infected animals (Fig. S4). Collectively, these data suggest that CD300f deficiency is protective against severe rickettsiosis *in vivo*.

### CD300f-expressing macrophages are involved in *Rickettsia* invasion *in vivo*

**Fig 5:**
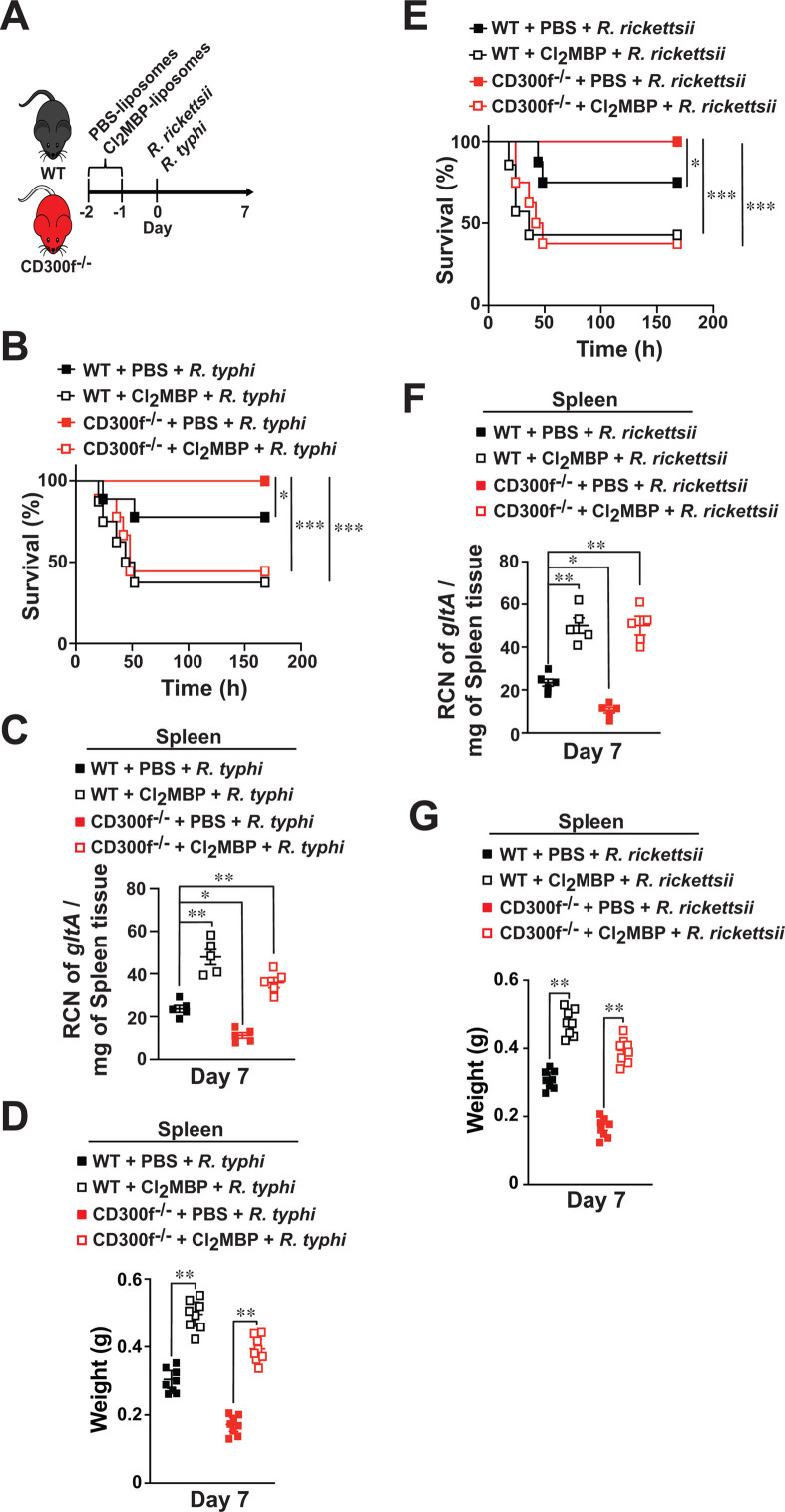
CD300f-expressing macrophages are involved in rickettsiae infection. (**A**) C57/B6J WT or CD300f^-/-^-deficient mice (*n* = 12 per experimental group) were i*.*v. injected twice (48 and 24 h) with PBS- or Cl_2_MBP-liposomes (200 µL/mouse) followed by i*.*v. injections with 10^5^ PFU of *R. typhi* (**B and D**) or *R. rickettsii* (**E–G**). Survival was monitored for 7 days (**B and E**). Bacterial burden (**C and F**) was determined in spleens of *Rickettsia*-injected WT or CD300f^-/-^ mice by RT-qPCR at day 7 (*n* = 5 for each treatment). RCN of *gltA* expression of rickettsiae was normalized by the expression of the housekeeping host gene, *GAPDH*. Spleen weights (**D and G**) from injected animals were evaluated at day 7 (*n* = 8). Error bars in panels C, D, F, and G represent the means ± SEM from five independent experiments. *P ≤ 0.05, **P ≤ 0.01, ***P ≤ 0.005.

Given the presented data and previous findings from others and our laboratory highlighting the importance of MΦ in controlling rickettsiae infection (22, 24, 31–34), we sought to determine the contributing role of MΦ in the observed susceptibility difference between *R. typhi*- or *R*. *rickettsii*-infected CD300f^-/-^ and WT mice. In this effort, we focused on both pathogenic rickettsiae, as infection studies using the non-pathogenic *R. montanensis* did not reveal any survival difference in our infection assays (Fig. 4A, C, and E). We injected C57/B6J WT and CD300f^-/-^-deficient mice via tail vein (i*.*v.) with liposome-encapsulated phosphate-buffered saline (PBS) or dichloromethylene bisphosphonate (Cl_2_MBP) at 48 and 24 h to deplete endogenous MΦ as described previously (20, 22). As our previous reports indicated that MΦ depletion caused an increase in susceptibility of animals toward rickettsiae infection (22), we performed our *in vivo* infection studies with a dose of either *R. typhi* or *R. rickettsii* that resulted in the development of a mild form of rickettsiosis (~LD_25_) in WT mice (Fig. 5A, B, and E). Our data further revealed that depletion of MΦ not only enhanced the mortality rate of *R. typhi*- and *R. rickettsii-*infected WT mice but also impaired the survival advantage observed in CD300f^-/-^ mice (Fig. 5B and E). In contrast, injection of PBS-liposomes did not alter the mortality rate of *R. typhi*- or *R. rickettsii*-infected WT or CD300f^-/-^ mice (Fig. 5B and E). Of note, the development of splenomegaly (represented by an increase in spleen weight) and consequently bacterial loads within the spleens were significantly elevated in rickettsiae-infected Cl_2_MBP-treated mice as compared to that of bacteria-infected PBS-liposomes-treated WT and CD300f^-/-^ animals (Fig. 5C and D, F and G). Taken together, our data suggest that the presence of CD300f-expressing MΦ plays a critical role in *Rickettsia* infection *in vivo*.

### Role of CD300f on macrophages in rickettsial pathogenesis *in vivo*

To further assess the role of CD300f-expressing MΦ in modulating rickettsiosis, we injected WT mice via tail vein (i*.*v.) with PBS- or Cl_2_MBP-liposome at 72 and 48 h prior to injections with either WT or CD300f^-/-^ BMDMΦ at a concentration of 5 × 10^6^ cells per mouse (Fig. S5A). Twenty-four hours later, the mice were injected (i*.*v.) with either *R. typhi* or *R. rickettsii* (10^5^ PFU) (Fig. S5B and E) to induce mild rickettsiosis (22).

Our data showed that the removal of MΦ not only enhanced the mortality rate of both *R. typhi*- and *R. rickettsii*-infected WT mice but also revealed that adoptively transferred BMDMΦ from WT mice partially rescued fatal rickettsiosis in Cl_2_MBP-treated WT mice (Fig. S5B and E). In addition, injection of BMDMΦ from CD300f^-/-^ mice into Cl_2_MBP-treated WT mice further reduced the mortality rate of *R. typhi*- or *R. rickettsii*-infected WT mice to levels seen in bacterial-infected PBS-liposomes-treated WT mice (Fig. S5B and E). Accordingly, the development of splenomegaly and bacterial loads within the spleens was significantly increased in bacteria-infected Cl_2_MBP-treated WT mice as compared to PBS-liposomes-treated WT mice (Fig. S5C and D, F and G). Adoptive transfer of WT BMDMΦ reduced the spleen weights and bacterial burdens in rickettsiae-infected Cl_2_MBP-treated WT mice (Fig. S5C and D, F and G). Moreover, injection of BMDMΦ from CD300f^-/-^ mice further reduced the spleen weights and bacterial burdens in Cl_2_MBP-treated WT mice, reaching the levels observed in rickettsiae-infected PBS-liposomes-treated WT mice (Fig. S5C and D, F and G). Of note, our adoptive transfer experiments did not completely recapitulate the protective phenotype observed in CD300f^-/-^ mice, which could be the result of not fully reestablishing the levels of endogenously expressed MΦ.

Collectively, these data suggest that (i) rickettsiae utilize the PS-CD300f ligand-receptor axis to gain access into MΦ, and (ii) CD300f-expressing MΦ are involved in rickettsial pathogenesis *in vivo*.

## DISCUSSION

Successful host invasion by intracellular bacteria requires receptor-ligand engagement and subversion of host endocytic bactericidal pathways (35, 36). However, research on how *Rickettsia* species invade and avoid cytosolic defense surveillance for host colonization is only now emerging beyond the difficulties posed by their obligate intracellular replication and a life cycle that involves arthropod and vertebrate hosts (4, 7). Intriguingly, *R. rickettsii* OmpA (37) does not impact the virulence in animal models, while *Rickettsia parkeri* OmpA (38) and Sca2 (39) were shown to impact virulence. The typhus group (TG) rickettsiae (e.g., *R. typhi*) express various surface cell antigens, including Sca1, Sca2, Sca3, Sca4, and Sca5, but do not express a functional gene for OmpA (*Sca*0), suggesting that OmpA is not required for virulence (7, 40, 41). In a recent report, we demonstrated that *R. typhi* OmpB (encoded by the *Sca5* gene) plays a role in host cell invasion (42); however, the functional importance of other Sca’s in TG rickettsiae invasion and virulence remains to be determined (7, 41, 43, 44). Of note, the role of *Rickettsia conorii* LPS in influencing virulence remains to be resolved (45, 46). Taken collectively, these reports suggest the presence of alternative or other available membrane components that could function as ligands for host receptors.

Previous research on endothelial cells, originally considered the main host target of rickettsiae, resulted in the identification of four probable host cell receptors (Ku70 [8], α2β1 [9], FGFR [10], and Epac [11]). However, selective targeting of those receptors showed only moderate inhibition of *Rickettsia* invasion, indicating cooperative and/or alternative receptor-ligand systems for bacteria engulfment. Furthermore, no receptor-ligand mechanism for rickettsial invasion has been identified for professional phagocytes like MΦ. To address this knowledge gap, we focused on receptor-ligand systems involved in efferocytosis, a mechanism required for cellular homeostasis, which is often hijacked by pathogens including viruses and bacteria to promote their host invasion (12–15, 36, 47). As this process involves the recognition of “eat-me” signals (e.g., phosphatidylserine [PS]) on apoptotic cells through various PS receptors including MerTK, TIM1/4, or CD300s (e.g., CD300f) (12–15, 36, 47), we leveraged the well-established *CD300f* knockout mouse model (19). In this study, we demonstrated that CD300f^-/-^ but not WT mice were protected against *R. typhi*- or *R. rickettsii*-induced fatal rickettsiosis. As previous reports from others and our laboratories suggest that rickettsiae exhibit species-specific immune modulatory responses in MΦ to establish an intracellular niche (22, 24, 31, 33, 34, 48–53), we next evaluated the role of CD300f on MΦ during rickettsial infection. *In vivo* depletion of endogenous MΦ not only increased the overall bacterial burden and the development of splenomegaly but also enhanced the mortality rate of *R. typhi*- or *R. rickettsii-*infected WT and CD300f^-/-^ mice. This suggests that the removal of MΦ enhances the susceptibility of animals toward rickettsiae infection, which is further supported by our previous finding (22). Intriguingly, our adoptive transfer studies demonstrated that reintroduction of WT, and to a greater extent CD300f^-/-^ BMDMΦ, can ameliorate the severity of rickettsiae infection (Fig. S5). In fact, the transfer of CD300f^-/-^ BMDMΦ almost recapitulates the protective phenotype observed in CD300f^-/-^ mice (Fig. S5). Taken together, these data indicate that CD300f-expressing MΦ play a crucial role in rickettsial infection *in vivo*.

Our study did not evaluate a potential contributing role of other CD300f-expressing cell types, including, among others, neutrophils or dendritic cells. CD300f has been shown to suppress the antimicrobial activity of neutrophils against *Pseudomonas aeruginosa* and *Candida albicans* infection (54). However, the precise role of neutrophils in controlling rickettsial growth in the host requires further clarification and will be addressed in the future (51, 55). Dendritic cells seem to mount different immune responses against *R. conorii* infection dependent on the *in vivo* model (protective type 1 response in C57BL/6 mice vs suppressive adaptive immunity in C3H/HeN animals) (56). Intriguingly, reports from other laboratories imply that in contrast to CD300f function on MΦ, its expression inhibited efferocytosis by dendritic cells (57), suggesting that CD300f might modulate rickettsiae infection and other cellular responses differently in dendritic cells as compared to MΦ; however, the precise mechanism remains to be investigated.

As the CD300 family is composed of several members, we evaluated the expression levels of *CD300a, CD300b*, *CD300c*, *CD300d*, and *CD300f* in BMDMΦ from both WT and CD300f^-/-^ mice, and demonstrated that the lack of CD300f expression did not affect the expression of any of the tested CD300s. In addition, we performed invasion assays using WT, CD300f^-/-^-, and CD300d^-/-^-deficient BMDMΦ, and showed that CD300f, and not CD300d, affected the internalization of *R. typhi*, *R. rickettsii*, and *R. montanensis*. Collectively, our findings indicate that CD300f, but not CD300d, plays a predominant role in facilitating rickettsiae invasion in MΦ. However, it is important to note that MΦ express additional PS-binding receptors, including, among others, Ku70 (58), CD300b (20, 25), Tim4 (59), and BAI1 (60), so it is plausible that CD300f can directly or indirectly cooperate with other receptors to modulate phagocytosis of their cargo, a mechanism described for Tim4 (61), and our future work will address these questions.

As CD300f-receptor engagement by rickettsiae would likely involve a ligand, we searched for putative ligands on the rickettsial outer membrane and found the presence of several glycerophospholipids (GPLs) (62, 63), including, among others, PS and phosphatidylglycerol (PG), known “eat-me” signals involved in efferocytosis (12–14, 47). Previous findings by others, along with our own work, have shown that obligate intracellular bacteria such as *Rickettsia* species possess most of the enzymes required for the biosynthesis of glycerophospholipids, such as PS. However, some metabolic gaps remain because of missing enzymes in the biosynthetic pathway, illustrating their dependence on host precursors to synthesize downstream metabolites (64–66). Based on these findings, we tested the hypothesis that *Rickettsia* engulfment is mediated by CD300f-PS systems generally used for efferocytosis. Our data revealed the importance of bacterial PS for engulfment in WT BMDMΦ by pre-incubating *R. typhi*, *R. rickettsii*, and *R. montanensis* with rAnxV, a molecule known to block PS-mediated efferocytosis (18, 19). To elucidate the mechanism of PS-CD300f-mediated engulfment of rickettsiae, we determined, using flow cytometry and IFA, that *R. typhi*, *R. rickettsii*, and *R. montanensis* express external and internal PS moieties. Furthermore, we addressed the potential PS carryover from host cell membrane remnants by performing invasion assays of all three *Rickettsia* using purified bacteria with different purities (PP, CP, and SP) and showed a similar internalization kinetics among all samples, suggesting that any potential PS carryover was not a contributing factor to the phagocytic process. Intriguingly, our data revealed differences in bacterial PS-surface expression between both the pathogenic *R. typhi* and *R. rickettsii* species and the non-pathogenic *R. montanensis* strain, indicating that surface-expressed PS might contribute to the observed dissimilarities in pathogenicity among *Rickettsia* species, which will be evaluated in our future work.

In addition, it is important to note that not all externalized PS is functionally equivalent. Therefore, it is still feasible that the PS found on the bacterial outer membrane is not directly involved in the phagocytosis process, which was described for other cells (e.g., monocytes and macrophages [67, 68]). It also remains plausible that the PS promoting *Rickettsia* engulfment is provided by a bystander apoptotic cell remnant, a mechanism described for *Trypanosoma brucei* (69), or via a PS-cloaking mechanism, a pathway described for *Listeria monocytogenes* (70). Although PS is considered a key physiological ligand for both human and mouse CD300f receptors (13, 18–20), recent findings indicate a subtle difference in ligand specificity dependent on the cellular context (71, 72). Thus, it is tempting to speculate that other ligands may contribute to the internalization process of rickettsiae, which will be addressed in our future work.

In sum, we now present a working model for both pathogenic and non-pathogenic rickettsiae that utilizes bacterial PS to bind to the efferocytic CD300f receptor on MΦ to facilitate host invasion.

## MATERIALS AND METHODS

### Animals

All experiments were conducted in a fully Association for Assessment and Accreditation of Laboratory Animal Care International (AAALAC)-accredited program using 8- to 10-wk-old female C57BL/6J WT, CD300d^−/−^, or CD300f^−/−^ mice in a specific-pathogen-free environment according to the University of Maryland School of Medicine Institutional Animal Care and Use Committee (IACUC protocol: AUP-00000110).

### Antibodies and reagents

Antibodies against whole *Rickettsia* (SFG or TG) were raised in-house (22, 24, 53). Lysozyme and anti-PS antibody were purchased from Sigma. ProLong Gold antifade mounting medium with 4′,6-diamidino-2-phenylindole (DAPI), paraformaldehyde (PFA), and Alexa 594- and 647-conjugated secondary antibodies, as well as Alexa Fluor 488-conjugated wheat germ agglutinin (WGA), were purchased from Thermo Fisher Scientific.

### Bacterial strains, cell culture, and infection

Vero76 cells (African green monkey kidney, ATCC, RL-1587) were maintained in minimal Dulbecco’s modified Eagle’s medium (DMEM) supplemented with 10% heat-inactivated fetal bovine serum (FBS) at 37°C with 5% CO_2_. *R. rickettsii* (*Sheila Smith*) strain was obtained from Dr. Ted Hackstadt (Rocky Mountain Laboratories, NIH, Hamilton, MT, USA), and *R. typhi* strain (Wilmington) was obtained from CDC. All *Rickettsia* species were propagated in Vero76 cells grown in DMEM supplemented with 5% FBS at 34°C with 5% CO_2_. Rickettsiae from infected host cells were purified as described previously (23, 29, 73, 74). Briefly, rickettsiae-infected host cells were disrupted by vortexing with 1 mm glass beads. The disrupted host cells were centrifuged at 250 × *g* for 5 min at 4°C to remove host cell debris or any remaining intact host cells, and the supernatant carrying released CP rickettsiae was collected. The supernatant carrying released rickettsiae (CP) was then centrifuged at 9,000 × *g* for 3 min at 4°C. The pellet was collected and washed in 1× PBS to obtain PP rickettsiae. The rickettsiae (PP) were resuspended in 1× PBS, layered on a 20% sucrose cushion at a 1:1 ratio, and centrifuged at 16,000 × *g* for 15 min at 4°C. The pellet was collected and washed in 1× PBS to obtain SP rickettsiae. For early stages of infection (before the doubling time [8 to 10 h] of rickettsiae), a higher multiplicity of infection (MOI) of 20 (e*.*g., 0.5 and 2 hours postinfection [hpi] for immunofluorescent assay) was used to ensure the presence of a sufficient number of bacteria, as compared to an MOI of 5 at later time points (24 hpi), to determine the biological functions of the bacteria during host infection (23, 24, 53, 75, 76). Tail vein injection (i*.*v.) of PP *Rickettsia* (10^5^–10^6^ PFU) resuspended in PBS was used to initiate infection in mice as described previously (22). Splenic tissue specimens were collected at the indicated times and were used for bacterial burden analysis by RT-qPCR described below.

### Differentiation of bone marrow-derived macrophages

Bone marrow cells were isolated from femurs and tibias of WT, CD300d^-/-^, or CD300f^-/-^ mice. Differentiation was induced by culturing bone marrow cells in RPMI 1640 medium supplemented with 10% FBS and 30% L929-conditioned medium (a source of macrophage colony-stimulating factor) and cultured for 7 days as described previously (20, 22, 24).

### RNA isolation and quantitative PCR

To determine viable bacterial number during the course of host infection, we performed RT-qPCR assay on isolated RNA (75, 77, 78). BMDMΦ samples were collected at different times of postinfection, while spleens were collected at day 7 postinfection. RNA was extracted from 1 × 10^6^ BMDMs or 100 µL of organ homogenate using the Quick-RNA miniprep kit (ZymoResearch). The iScript Reverse Transcription Supermix kit (Bio-Rad) was used to synthesize cDNAs from 200 ng of RNA according to the manufacturer’s instructions. The qPCR amplification and detection were performed on a QuantStudio 3 Real-Time PCR System (Applied Biosystems by Thermo Fisher) as described previously (22, 24, 79). Briefly, qPCR was carried out using SYBR Green (Thermo Fisher Scientific), 2 µL cDNA, and 1 µM each of the following oligonucleotides for rickettsial (housekeeping) citrate synthase gene (*gltA*) and host (housekeeping) *GAPDH* gene. Oligonucleotides for detecting murine *CD300a*, *CD300b*, *CD300c*, *CD300d*, and *CD300f* were obtained from Qiagen. Cycling conditions were as follows: 1 cycle at 95°C for 3 min; 40 cycles at 95°C for 15 sec, 55°C for 15 sec, and 72°C for 20 sec; and 1 cycle to generate the dissociation curve. Melting curve analyses were performed at the end of each run to ensure that only one product was amplified. The relative copy number (RCN) of *gltA* expression was normalized by the expression of *GAPDH* and was calculated with the equation: RCN = E^−ΔCt^, where E = efficiency of PCR, and Ct = Ct *target* − Ct *GAPDH*, as described previously (22, 24).

### *In vivo* depletion of macrophages following rickettsiae challenge

C57/B6J WT or CD300f^-/-^ mice were injected via tail vein (i*.*v.) using liposome-encapsulated PBS or dichloromethylene bisphosphonate (Cl_2_MBP), as described previously (20, 22). Mice were injected twice (48 and 24 h) with PBS- or Cl_2_MBP-liposomes (200 µL/mouse), followed by injections (i*.*v.) 24 h later with 10^5^ PFU of *R. typhi* or *R. rickettsii*. Survival was monitored for 7 days. Bacterial burden was determined in spleens of uninfected or *R. typhi*- and *R. rickettsii*-injected mice by RT-qPCR at day 7 (*n* = 5 for each treatment).

### Adoptive transfer of bone marrow-derived macrophages following *Rickettsia* challenge

C57/B6J WT mice were injected via tail vein (i*.*v.). Mice were injected twice (72 and 48 h) with PBS- or Cl_2_MBP-liposomes (200 µL/mouse), followed by injections with BMDMΦ (5 × 10^6^ cells/mouse) from CD300f^-/-^ or WT mice, as described previously (20, 22). Twenty-four hours later, the mice were injected (i*.*v.) with 10^5^ PFU of *R. typhi* or *R. rickettsii*. Survival was monitored for 7 days, and bacterial burden was determined in spleens of uninfected or *Rickettsia*-injected mice by RT-qPCR.

### Immunofluorescent assay (IFA)

Eight-well chamber slides were seeded with WT, CD300d^-/-^, or CD300f^-/-^ BMDMΦ (~50 × 10^4^ cells/well) and were infected using purified *Rickettsia* species (MOI = 20 [0.5 and 2 h] or 5 [24 h]) as described previously (22–24, 53). Briefly, rickettsiae were added to BMDMΦ and incubated for various lengths of time at 34°C. Following incubation, cells were washed three times with 1× PBS and fixed with 4% PFA for 20 min at room temperature. For IFA, cells were stained with the following primary Abs: anti-*Rickettsia* (SFG [1:100 dilution]; TG [1:500 dilution]), anti-PS or anti-IgG control Abs (1:100 dilution), and Alexa Fluor 488-conjugated WGA, as described previously (23). Cells were then washed with 1× PBS and incubated for 1 h with anti-Alexa Fluor 594 and anti-Alexa Fluor 647 Abs diluted 1:1,500 in Ab-dilution buffer. Next, cells were washed with 1× PBS and mounted with ProLong Gold antifade mounting medium containing DAPI. Images were acquired using the Nikon W-1 spinning disk confocal microscope (University of Maryland Baltimore, Confocal Core Facility). Co-localization strength between *Rickettsia* and PS was analyzed by using the Fiji software as described previously (22, 24, 53). The percentage of internalized bacteria (approximately 200 bacteria were counted per strain and time point) was calculated by dividing the number of extracellular bacteria by the total number of bacteria, multiplying by 100, and then subtracting this number from 100% to get the percentage of intracellular bacteria.

For infection assays using rAnxV protein, partially purified *Rickettsia* was incubated with various concentrations of regular or heat-inactivated rAnxV for 0.5 h at 4°C. Rickettsiae treated with rAnxV were used to infect BMDMΦ for an additional 2 h at 34°C. Following incubation, cells were washed, fixed, and stained with anti-*Rickettsia* Abs as described above.

### Detection of phosphatidylserine by flow cytometry

Partially purified rickettsiae (~1 × 10^7^) were fixed in 70% ethanol (vol/vol) under constant mixing. Bacteria were centrifuged, and the pellet was resuspended in 1× PBS containing lysozyme (100 mg/L). Samples were incubated for 1 h at 37°C and were filtered using a nylon mesh. Bacteria were stained using an Annexin V apoptosis detection kit (BioLegend) following the manufacturer’s instructions. Briefly, bacteria were resuspended in an AnxV-dilution buffer at 1:10 and were incubated with APC-conjugated Annexin V for 30 min in the dark on ice. Samples were washed, resuspended in 1× PBS buffer, and acquired using a Becton-Dickinson Accuri C6 machine. Samples were analyzed by the FlowJo software (version 10).

### Statistical analysis

Data sets were considered statistically significant when a *P* ≤ 0.05 value was obtained by unpaired Student’s *t*-test (two tailed), paired Student’s *t*-test (two tailed), a one-way analysis of variance (ANOVA) with either multiple comparisons or comparison to WT bacteria, a two-way ANOVA, or a log-rank (Mantel-Cox) test. Statistical analyses were performed using the GraphPad Prism Software, version 8, and samples were denoted using the following asterisks: *P ≤  0.05, **P ≤  0.01, ***P ≤  0.005, ****P ≤  0.001. Data are presented as mean ± standard error of the mean (SEM), unless stated otherwise.

## SUPPLEMENTAL MATERIAL

Fig. S1 iai.00059-25-s0001.pdf CD300f is highly expressed and plays a key role on macrophages to modulate rickettsiae invasion *in vitro*.

Fig. S2 iai.00059-25-s0002.eps Anti-phosphatidylserine antibody specificity staining in purified rickettsiae.

Fig. S3 iai.00059-25-s0003.pdf Assessment of PS-carryover from different Rickettsia–host cell preparations.

Fig. S4 iai.00059-25-s0004.pdf Splenic data during infection of pathogenic and non-pathogenic Rickettsia species.

Fig. S5 iai.00059-25-s0005.pdf CD300f-expressing macrophages are involved in rickettsial pathogenesis *in vivo*.

ASM does not own the copyrights to Supplemental Material that may be linked to, or accessed through, an article. The authors have granted ASM a non-exclusive, world-wide license to publish the Supplemental Material files. Please contact the corresponding author directly for reuse.
