# Supplementary material for: Phosphatidylserine-binding receptor, CD300f, on macrophages mediates host invasion of pathogenic and non-pathogenic rickettsiae
Source: Infect Immun. 2025 May 1;93(6):e00059-25. doi: 10.1128/iai.00059-25 (PMC12150758; doi:10.1128/iai.00059-25)
Supplement: Fig. S1 — CD300f is highly expressed and plays a key role on macrophages to modulate rickettsiae invasion in vitro. [file iai.00059-25-s0001.pdf]

Fig. S1

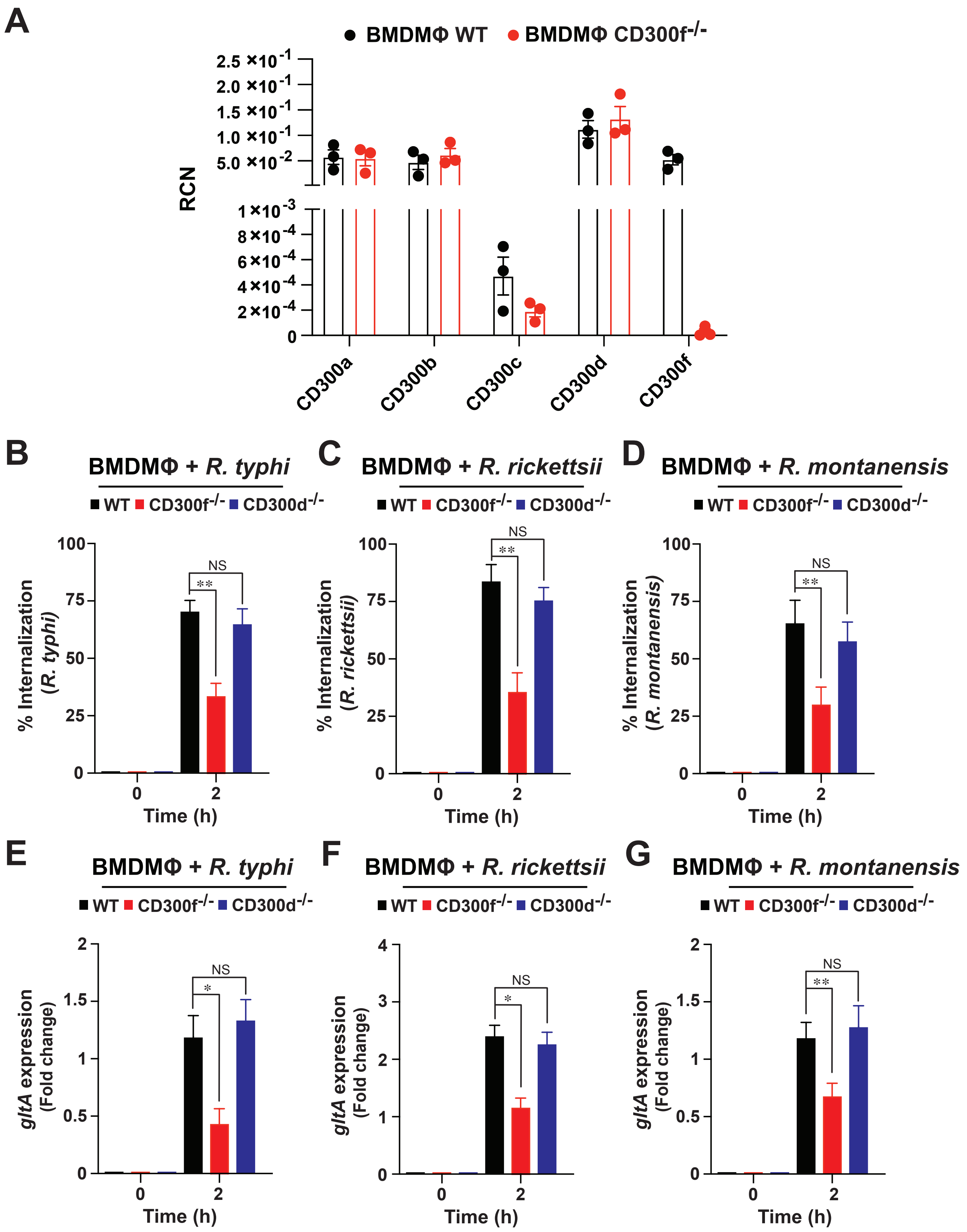

Fig. S1. CD300f, is highly expressed and plays a key role on macrophages to modulate rickettsiae invasion *in vitro*. (A) Expression levels of murine *CD300a*, *CD300b*, *CD300c*, *CD300d*, *CD300f* were assessed in uninfected WT and CD300f$^{-/-}$ BMDMΦ by RT-qPCR (n = 3 per group). Expression levels of all *CD300s* was normalized by *GAPDH* expression. (B-D) Rickettsial invasion was monitored by IFA at 2 hpi as described previously (23). (E-G) Bacterial burden in WT and CD300f$^{-/-}$ BMDMΦ infected with *R. typhi*, *R. rickettsii*, or *R. montanensis* was determined at 2 hpi (n = 5 per group) by RT-qPCR. RCN of *gltA* expression of rickettsiae was normalized by the expression of the housekeeping host gene, *GAPDH*. Error bars in panels A-G represent means ± SEM from 3-5 independent experiments. NS, nonsignificant; *$P \leq 0.05$; **$P \leq 0.01$.
