# Supplementary material for: Phosphatidylserine-binding receptor, CD300f, on macrophages mediates host invasion of pathogenic and non-pathogenic rickettsiae
Source: Infect Immun. 2025 May 1;93(6):e00059-25. doi: 10.1128/iai.00059-25 (PMC12150758; doi:10.1128/iai.00059-25)
Supplement: Fig. S3 — Assessment of PS-carryover from different Rickettsia–host cell preparations. [file iai.00059-25-s0003.pdf]

Fig. S3

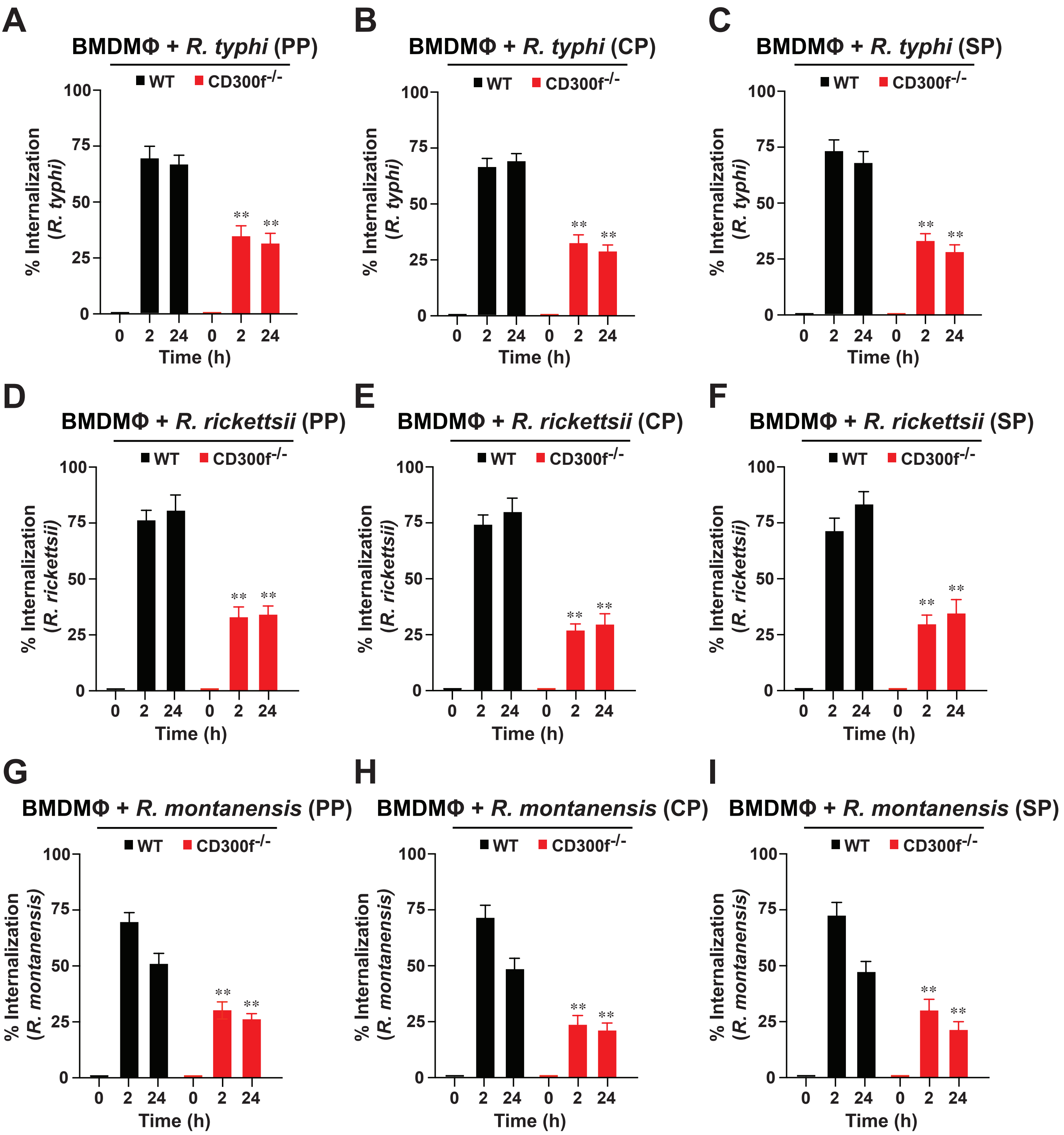

Fig. S3. Assessment of PS-carryover from different *Rickettsia*-Host cell preparations. Three different types of inocula [partially purified (PP), crudely purified (CP), and sucrose purified (SP)] from *R. typhi* (A-C), *R. rickettsii* (D-F), or *R. montanensis* (H-I) were utilized to infect WT or CD300f$^{-/-}$ BMDMΦ for 2 and 24 hpi using a MOI of 20 (2 hpi) and 5 (24 hpi) respectively. Rickettsial invasion was monitored by IFA as described previously (23). Error bars in panels A-I represent means ± SEM from 5 independent experiments. NS, nonsignificant; $^{**}P \leq 0.01$.
