# Supplementary material for: Phosphatidylserine-binding receptor, CD300f, on macrophages mediates host invasion of pathogenic and non-pathogenic rickettsiae
Source: Infect Immun. 2025 May 1;93(6):e00059-25. doi: 10.1128/iai.00059-25 (PMC12150758; doi:10.1128/iai.00059-25)
Supplement: Fig. S4 — Splenic data during infection of pathogenic and non-pathogenic Rickettsia species. [file iai.00059-25-s0004.pdf]

Fig. S4

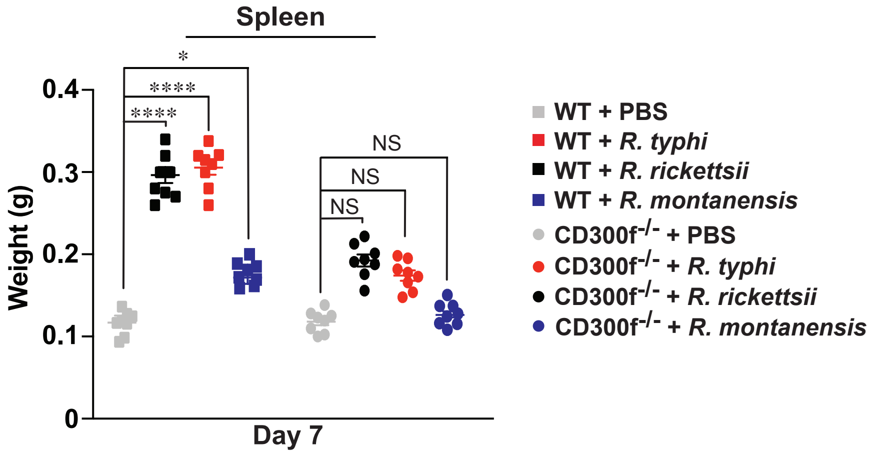

Fig. S4. Splenic data during infection of pathogenic and non-pathogenic *Rickettsia* species. C57BL/6J WT and CD300f$^{-/-}$ mice injected (i.v.) with *R. typhi*, *R. rickettsii*, *R. montanensis*, and PBS (dose of 10^6 PFU). Spleen weights from injected animals were evaluated at day 7 (n = 8). Error bars represent means ± SEM from five independent experiments. NS, nonsignificant; **P* ≤ 0.05; *****P* ≤ 0.001.
