# Supplementary material for: Phosphatidylserine-binding receptor, CD300f, on macrophages mediates host invasion of pathogenic and non-pathogenic rickettsiae
Source: Infect Immun. 2025 May 1;93(6):e00059-25. doi: 10.1128/iai.00059-25 (PMC12150758; doi:10.1128/iai.00059-25)
Supplement: Fig. S5 — CD300f-expressing macrophages are involved in rickettsial pathogenesis in vivo. [file iai.00059-25-s0005.pdf]

Fig. S5

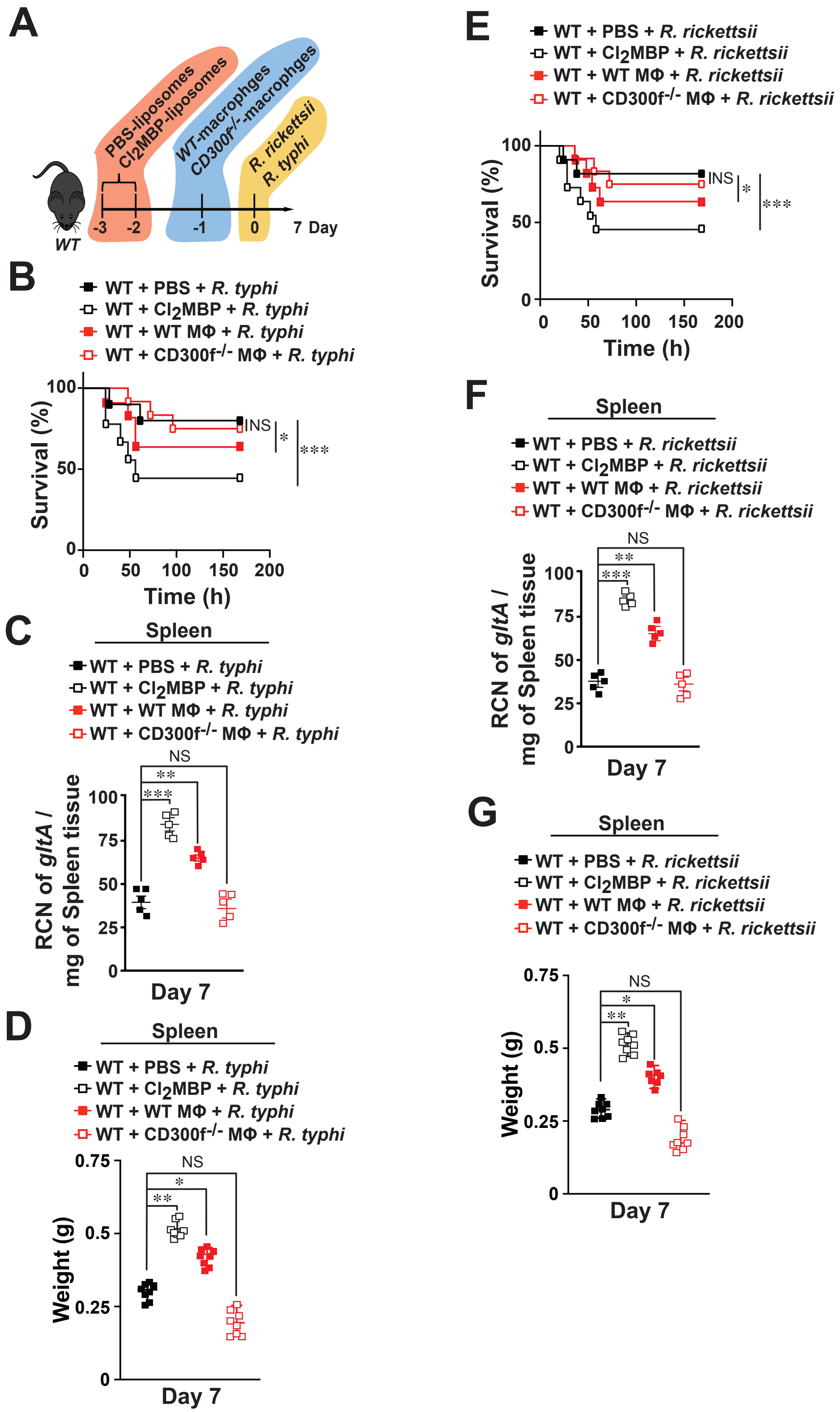

Fig. S5. CD300f-expressing macrophages are involved in rickettsial pathogenesis *in vivo*. (A) C57/B6J WT mice (n = 12 per experimental group) were injected via tail vein (i.v.) using liposome-encapsulated PBS- or dichloromethylene bisphosphonate (Cl_2MBP). Mice were injected twice (72 and 48 h) with PBS- or Cl_2MBP-liposomes (200 μl/per mouse) followed by injections with BMDMΦ (5 x 10^6 cells/mouse) from CD300f$^{-/-}$, or WT mice. Twenty-four hours later, mice were injected (i.v.) with 10^5 PFU of *R. typhi* (B-D) or *R. rickettsii* (E-G). Survival was monitored for 7 days (B and E). Bacterial burden (C and F) was determined in spleens of *Rickettsia*-injected WT or CD300f$^{-/-}$ mice by RT-qPCR at day 7 (n = 5 for each treatment). RCN of *gltA* expression of rickettsiae was normalized by the expression of the housekeeping host gene, *GAPDH*. Spleen weights (D and G) from injected animals were evaluated at day 7 day (n = 8). Error bars in panels C, D, F, and G represent means ± SEM from five independent experiments; NS, non-significant; *$P \leq 0.05$; **$P \leq 0.01$; ***$P \leq 0.005$.
